# Dose–exposure–efficacy response of intravenous immunoglobulin G 10% in multifocal motor neuropathy

**DOI:** 10.1002/acn3.52098

**Published:** 2024-07-08

**Authors:** Zhaoyang Li, Stefan Roepcke, Ryan Franke, Leman Yel

**Affiliations:** ^1^ Clinical Pharmacology & Early Clinical Development Takeda Development Center Americas, Inc. Cambridge Massachusetts USA; ^2^ Pharmacometrics Cognigen, a division of Simulations Plus Buffalo New York USA; ^3^ Quantitative Clinical Pharmacology Cognigen, a division of Simulations Plus Buffalo New York USA; ^4^ Clinical Medicine Takeda Development Center Americas, Inc. Cambridge Massachusetts USA; ^5^ Present address: Clinical Pharmacology Pfizer, Inc. 10777 Science Center Drive San Diego 92121 California USA; ^6^ Present address: Department of Medicine University of California Irvine California USA

## Abstract

**Objective:**

Multifocal motor neuropathy is a rare chronic immune‐mediated neuropathy with impaired grip strength representing a common symptom. While intravenous immunoglobulin G is an effective treatment for the disease, significant variation in treatment response has been observed but not well understood. This analysis characterized dose–exposure–response relationships in multifocal motor neuropathy, using grip strength as a clinical efficacy measure.

**Methods:**

Serum immunoglobulin G trough concentrations and grip strength data for the more affected hand from a Phase 3, randomized, double‐blind, placebo‐controlled, crossover trial of intravenous immunoglobulin 10% in 44 patients with multifocal motor neuropathy (NCT00666263) were used to develop a population pharmacokinetic–pharmacodynamic model.

**Results:**

The model adequately described the observed pharmacokinetic and pharmacodynamic data and relationships between intravenous immunoglobulin 10% dose, serum immunoglobulin G trough levels, grip strength, and inter‐patient variabilities in multifocal motor neuropathy. Model‐based simulations for various dosing regimens (0.4–2.0 g/kg every 2–4 weeks) indicated that ≥1.6 g/kg/month would achieve clinically meaningful improvements in grip strength (≥4 kg) in ≥70% of patients. More frequent dosing at an equivalent monthly dose led to a more consistent response in grip strength. Furthermore, splitting the dose over multiple days for high doses (>1 g/kg) did not impact grip strength.

**Interpretation:**

These findings suggest that the majority of patients with multifocal motor neuropathy would respond rapidly to intravenous immunoglobulin 10% with a range of dosing regimens. Shorter dosing intervals may avoid the diminishing response seen with longer dosing intervals. Dose‐splitting provided similar outcomes while offering flexibility and convenience.

## Introduction

Multifocal motor neuropathy (MMN) is a rare, chronic, immune‐mediated neuropathy which is estimated to affect less than one individual in every 100,000 worldwide.^1^, ^2^ MMN results in progressive, asymmetric distal limb weakness (most commonly affecting the arms), with slow and gradual symptom progression leading to substantial functional disability that can interfere with simple daily activities, such as writing, washing, or dressing.^1^, ^2^, ^3^, ^4^, ^5^ One of the most common symptoms of MMN is impaired grip strength,^4^ which is often used as a measure of efficacy in randomized clinical trials of potential therapies for the disease.^1^, ^6^

Intravenous immunoglobulin (IVIG) 10% has been shown to be an effective treatment for MMN, and is currently approved in the USA and the EU for use as induction and maintenance therapy to improve muscle strength and reduce limb disability in affected adults.^7^, ^8^ Substantial variations in response to immunoglobulin treatment have been observed,^2^ and the recommended IVIG dose and infusion regimen varies from patient to patient based on their body weight and clinical status.^7^, ^8^ Owing to the rarity of the condition, current clinical research into immunoglobulin therapies in MMN is limited to small studies (typically *n* < 20), primarily aimed at ascertaining the optimal IVIG maintenance dosing regimens for individual patients with MMN, evaluating empirical responses (e.g., impact on grip strength) and assessing the safety and long‐term efficacy of subcutaneous immunoglobulin treatments containing hyaluronidase.^9^, ^10^, ^11^, ^12^, ^13^ As such, little is known regarding the interplay between IVIG 10% dose, serum immunoglobulin G (IgG) exposure, and clinical efficacy in MMN, as well as patient characteristics that may impact treatment response.^6^, ^14^, ^15^, ^16^ Therefore, it is imperative to conduct further research to understand the dose–exposure–efficacy response relationship of immunoglobulin therapies in MMN, and to optimize individualized treatment and dosing with respect to potential patient factors.^6^, ^14^, ^15^, ^16^ The use of reliable and responsive outcome measures in such investigations is important in quantifying clinically relevant changes in response to changing IVIG dose. Grip strength is a key diagnostic criterion in MMN that is widely used as an outcome in clinical trials and is included in several modern combined measures of disability in the disease.^17^ Characterization of the relationships between IVIG 10% dose, serum IgG concentrations, and grip strength (as an indicator of clinical efficacy) in MMN could help support future trial design and aid the selection of doses and treatment regimens in wider patient populations.^18^, ^19^

Pharmacokinetic (PK) and pharmacodynamic (PD) modeling has been an integral part of drug development for many years, and is used to determine appropriate doses and to optimize dosing regimens over the course of clinical development.^20^ For IgG, there have been multiple studies that have successfully employed PK modeling to simulate IgG levels following interventions in patients with primary immunodeficiencies, enabling the optimization of dosing regimens and the identification of intrinsic and extrinsic factors that may significantly affect IgG levels following treatment.^21^, ^22^, ^23^, ^24^, ^25^ Modeling studies have also been used to assess high‐dose IVIG treatment, and have explored IgG pharmacokinetics in Guillain–Barré syndrome and the relationship between IgG exposure and disease severity in chronic inflammatory demyelinating polyradiculoneuropathy (CIDP), supporting the implementation of personalized therapy for patients with these conditions.^26^, ^27^ However, no modeling studies have yet been published describing the relationship between IVIG dose, serum IgG exposure, and clinical responses in patients with MMN.

A population PK model describing IgG PK following IVIG 10% administration in patients with MMN has been developed previously.^28^ The aim of the current analysis was to build upon this work and characterize the dose–exposure–efficacy response relationship following administration of IVIG 10% or placebo in patients with MMN, using population PK–PD modeling and grip strength as a measure of clinical efficacy. The effect of intrinsic and extrinsic patient factors as potential covariates on the PK–PD relationships was also assessed.

## Methods and Materials

### Data sources and handling

Serum trough IgG concentrations and grip strength measurements (for the more affected hand) were sourced from a Phase 3, randomized, double‐blind, placebo‐controlled, crossover trial of IVIG 10% (Gammagard; Baxalta US Inc., a member of the Takeda group of companies, Lexington, MA, USA/Kiovig; Takeda Manufacturing Austria AG, Vienna, Austria^7^, ^8^) in 44 adult patients with MMN (NCT00666263).^1^ Full details of the study methodology and clinical results have been published previously by Hahn et al.^1^ Briefly, eligible patients had a diagnosis of definite or probable MMN and had been on a stable IVIG 10% regimen for at least 3 months, at a stable dose of 0.4–2.0 g/kg body weight every 2–5 weeks as required for individual patients.^1^ After an initial stabilization period on open‐label IVIG 10%, patients were then randomized (1:1) to one of two treatment sequences of 12 weeks of IVIG 10% or placebo, followed by a second stabilization period on open‐label IVIG 10% to avoid carryover effects.^1^ This was then followed by another 12‐week crossover period of placebo or IVIG 10%, respectively, and a final stabilization period (open‐label IVIG 10%).^1^ During the 60‐week study, dosing regimens for IVIG 10% ranged 0.4–2.0 g/kg/infusion cycle, divided over one to five consecutive days, with a once every 2‐, 3‐, or 4‐weekly dosing cycle (Q2W, Q3W, or Q4W, respectively).^1^

For the PK–PD analysis dataset, serum total IgG concentrations, dosing/treatment information, demographics, grip strength measurements (as a measure of efficacy), clinical laboratory values, and other covariate information were extracted for individual patients. During the study, seven protocol‐specified grip strength assessments were performed using a DynEx digital dynamometer (MD Systems, Reynoldsburg, OH, USA; measurements reported in kilograms).^29^ These assessments were conducted at the start of the initial stabilization period, 7–12 days before collection of trough IgG samples at the end of each stabilization and crossover period, and at the end of the study. The grip strength of each hand was measured twice in triplicate and the mean grip strength calculated for each hand, with the more affected hand identified for use in the analysis. If the grip strength of a patient's more affected hand decreased by ≥50%, or if their physical strength decreased to the point of causing unacceptable difficulty in daily activities during any of the crossover periods, the patient was permitted to switch to the next stabilization phase of open‐label IVIG 10% without breaking the blind (referred to as accelerated switch). For patients who underwent an accelerated switch during either of the blinded crossover periods, serum trough IgG PK measurements were collected prior to treatment with open‐label IVIG 10%.

### Exploratory analysis of serum IgG exposure–grip strength correlation

Prior to modeling, an exploratory exposure–response analysis was performed to evaluate correlations between changes in grip strength and in serum trough IgG levels. Changes in grip strength between IVIG 10% treatment periods and placebo periods were analyzed using analysis of variance, and correlations between changes in trough IgG levels and in grip strength were analyzed using linear regression, with change in trough IgG as the independent variable and change in grip strength as the dependent variable.^18^

### Population PK–PD modeling and simulations

Grip strength over time and trough serum IgG concentration–time data were analyzed using a nonlinear mixed‐effects modeling approach using NONMEM version 7.3.0 (ICON, Hanover, NH, USA).^30^ Additional details are provided in the Supplementary Methods.

The final population PK–PD model was used to predict grip strength profiles and IgG concentrations under different IVIG 10% dosing regimens over 6 months of continuous treatment using Monte Carlo simulations. A virtual population (*n* = 1000) for each dosing scenario was created by randomly resampling from the 44 patients included in the modeling dataset. The dosing scenarios evaluated were: 0.4, 0.8, and 1 g/kg Q2W; 1 and 2 g/kg Q3W; 0.4, 0.8, 1, and 2 g/kg Q4W; and 2 g/kg Q4W split into four daily infusions. Treatment effect was assessed as the change in grip strength between active treatment and placebo. An increase in grip strength of 4 kg was defined as the minimum clinically meaningful improvement and used as a cutoff point based on prior analysis.^29^, ^31^, ^32^

## Results

### Analysis dataset and patient population

The final analysis dataset comprised 309 serum IgG concentration records from 44 patients.^18^, ^19^ From these patient records, a total of 573 grip strength measurements were included in the final analysis dataset. Baseline clinical covariates for the total PK–PD analysis population have been presented previously.^28^ In brief, patients had a mean (standard deviation [SD]) age of 51.7 (10.3) years, a body mass index of 27.9 (4.1) kg/m^2^ and 72.7% of patients were male. The mean (SD) total IgG dose was 1.0 (0.5) g/kg, with administration Q4W most commonly reported (61.4% of patients). At study entry, the grip strength of the more affected hand varied widely, ranging 1.2–44.8 kg (median = 14.9 kg; Table 1), as did the grip strength of the less affected hand (range: 3.2–51.3 kg; median = 27.8 kg).

**Table 1:** Grip strength measures for the more affected hand at baseline.

Grip strength (GS) measure	Treatment sequence 1 (IVIG then placebo)^a^ (*n* = 22)	Treatment sequence 2 (placebo then IVIG)^a^ (*n* = 22)	Overall (*n* = 44)
GS at study entry, kg			
Mean (SD)	19.6 (12.3)	14.4 (9.7)	17.0 (11.3)
Median (min, max)	18.1 (1.2, 44.8)	13.2 (3.1, 36.3)	14.9 (1.2, 44.8)
GS_AT_, kg			
Mean (SD)	21.1 (13.2)	17.5 (10.7)	19.3 (12.1)
Median (min, max)	19.4 (1.2, 45.2)	15.0 (3.0, 36.5)	15.0 (1.2, 45.2)
GS_PBO_, kg			
Mean (SD)	15.9 (10.9)	11.9 (9.0)	14.0 (10.1)
Median (min, max)	12.1 (3.1, 40.0)	9.9 (0.8, 28.3)	11.3 (0.8, 40.0)
ΔGS, kg			
Mean (SD)	−5.6 (6.6)	−6.0 (6.0)	−5.8 (6.2)
Median (min, max)	−4.7 (−20.5, 2.4)	−4.1 (−21.2, 0.2)	−4.5 (−21.2, 2.4)

GS_AT_, grip strength of the more affected hand during active treatment before the placebo period; GS_PBO_, grip strength of the more affected hand during placebo period; IVIG, intravenous immunoglobulin G; SD, standard deviation; ΔGS, difference in grip strength of the more affected hand between the placebo period and the active treatment before the placebo period.

^a^ Treatment sequences refer to active treatment (IVIG or placebo) during the crossover phases.

### Exploratory analysis of serum IgG exposure–grip strength correlation

**Figure 1:**
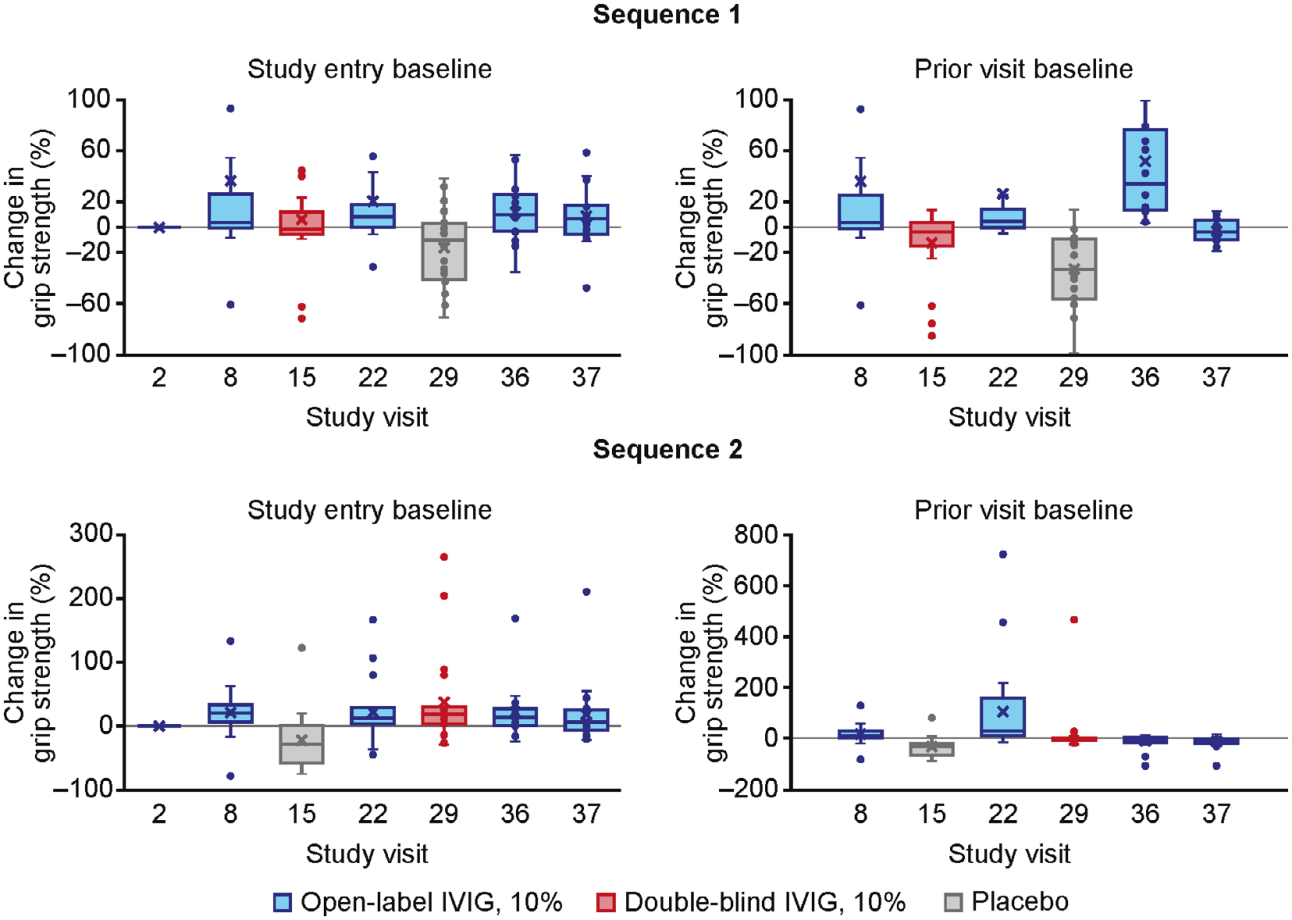
Change in grip strength by study visit (exploratory analysis).^18^ Percent change in grip strength in the more affected hand was calculated relative to either the baseline value at study entry or at the prior study visit (left and right panels, respectively). IVIG, intravenous immunoglobulin.

An exploratory exposure–response analysis showed that in patients switching from IVIG 10% to placebo, grip strength rapidly declined in the more affected hand (mean change: −28.7% and − 27.1% for treatment sequences 1 and 2, respectively; Fig. 1).^18^ In contrast, after blinded IVIG 10% treatment over the same period, the mean changes in grip strength from baseline for treatment sequences 1 and 2 were − 12.5% and + 2.1%, respectively (Fig. 1).^18^ The least‐square means of percent change in grip strength in the more affected hand significantly differed between IVIG 10% and placebo periods (22.6%; *p* < 0.001).^18^

**Figure 2:**
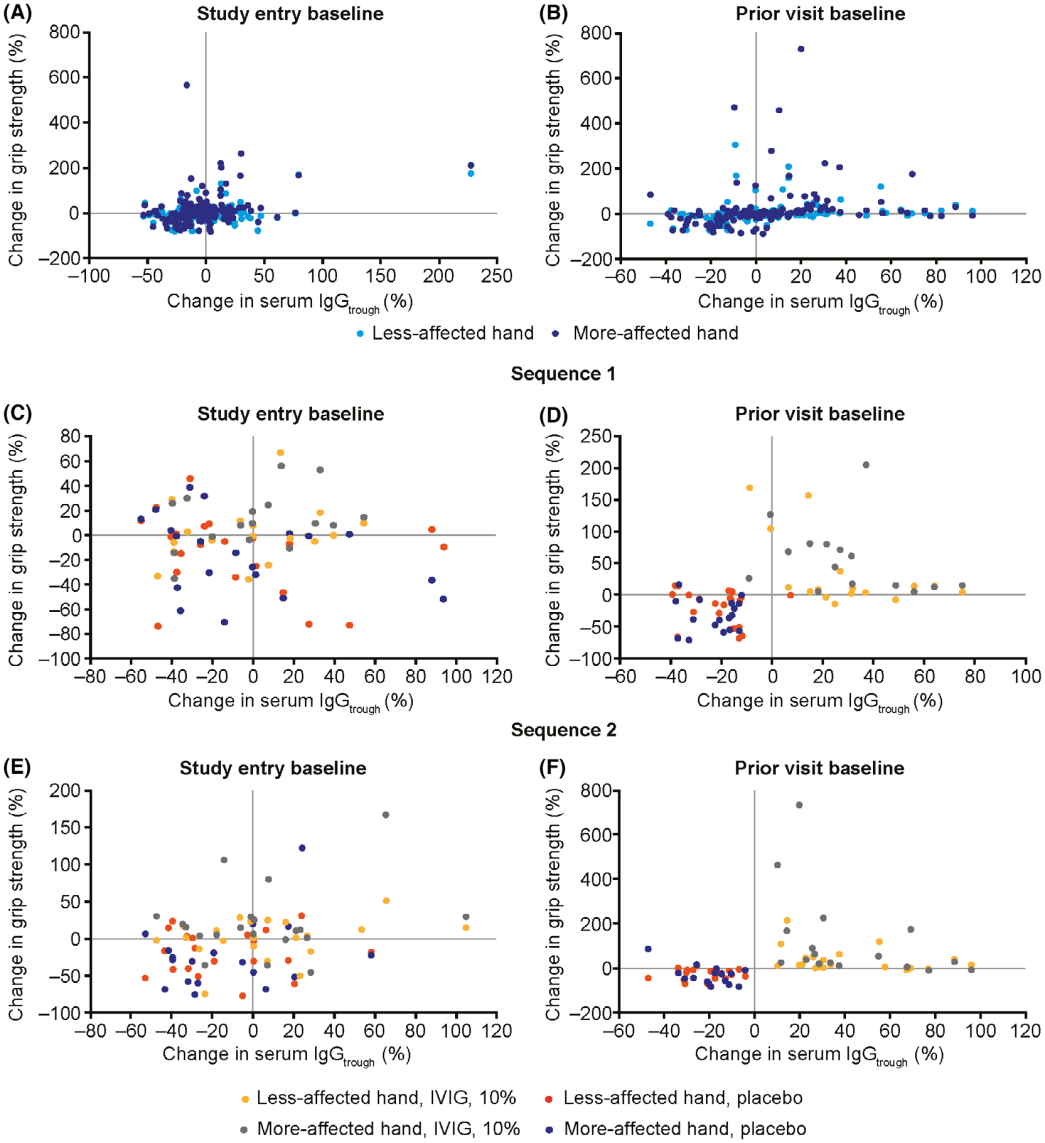
Change in grip strength versus change in serum IgG trough concentration (exploratory analysis).^18^ Percent changes in grip strength or serum IgG_trough_ were calculated relative to either the baseline value at study entry or at the prior study visit (left and right panels, respectively). Comparisons are shown at the group level across all time points (panels A and B) and the individual level during treatment switches for treatment sequence 1 (panels C and D) and sequence 2 (panels E and F). IgG, immunoglobulin G; IgG_trough_, trough immunoglobulin G concentration; IVIG, intravenous immunoglobulin.

When the relationship between change in grip strength and change in serum trough IgG level was examined, there were no correlations between percent change in grip strength and either serum trough IgG level (data not shown), or percentage change in serum IgG trough level across all time points, when using study entry as the comparison baseline (Fig. 2, left panels).^18^ However, when the prior study visit (defined as the visit immediately before the visit of interest) rather than study entry was used as the comparison baseline, percentage changes in grip strength for the more affected hand were positively correlated with percentage changes in serum IgG (Fig. 2, right panels).^18^ There were two distinct cohorts of patients: those with increased grip strength and IgG trough concentrations, and those with decreased grip strength and IgG trough concentrations. Similar results were also observed for the less affected hand.^18^

### Population PK–PD analysis

The final population PK–PD model took into account endogenous IgG production, IgG elimination, steady‐state IgG concentration in the absence of treatment (CBASE), and the inhibitory effect of IgG concentration on deterioration (DTR) of grip strength (Fig. S1). Interindividual variability in grip strength in the absence of treatment (GBASE) was included, in addition to the interindividual variability terms on the central volume of distribution (V1) and CBASE from the original population PK model.^28^ No significant covariates were identified.

**Figure 3:**
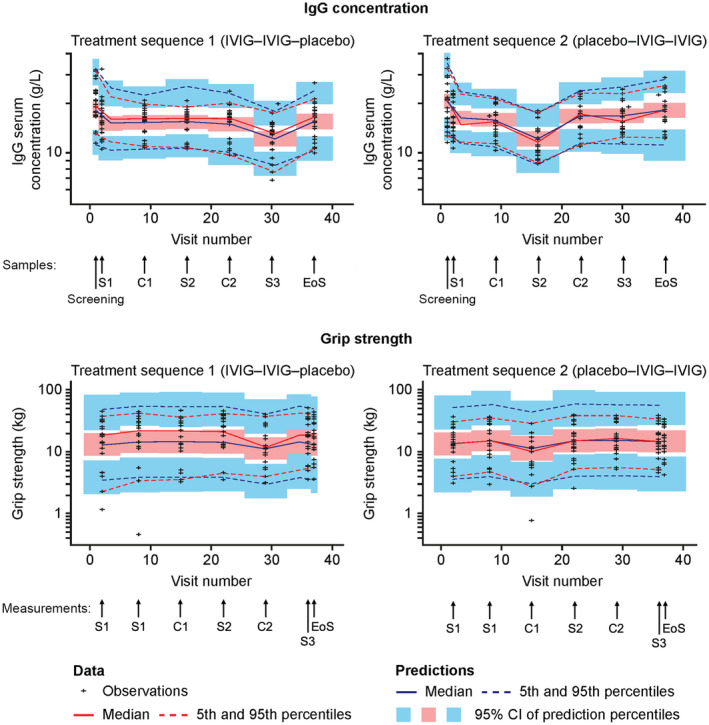
Simulation‐based VPCs for the final population PK–PD model, stratified by treatment sequence. The treatment sequence in the study arms is designated with IVIG–IVIG–placebo or placebo–IVIG–IVIG where IVIG (or placebo) at the first place refers to active treatment (or placebo) in the first crossover phase, IVIG in the second place refers to active treatment in the stabilization phase and IVIG (or placebo) in the third place refers to active treatment (or placebo) in the second crossover phase. Arrows beneath the x‐axes of the top panels indicate the IgG samples taken at screening, end of study, and at the start of each stabilization (S1, S2, and S3) and crossover (C1 and C2) phase. Arrows beneath the x‐axes of the bottom panels indicate the grip strength measurements taken at the end of each stabilization and crossover phase, at the start of S1 and at the end of the study. Medians and percentiles are plotted at the midpoint of each visit number interval. C1/C2, crossover phases; CI, confidence interval; EoS, end of study; IgG, immunoglobulin G; IVIG, intravenous immunoglobulin; PK–PD, pharmacokinetic–pharmacodynamic; S1/S2/S3, stabilization phases; VPC, visual predictive check.

The model was shown to adequately describe the observed data for grip strength and serum IgG trough levels, as shown by the goodness‐of‐fit plots (Fig. S2) and visual predictive checks (VPCs), stratified by treatment sequence (Fig. 3). Fixed‐effect parameters were estimated with acceptable precision (Supplementary Table 1). While there was a tendency for the model to slightly overpredict high grip strength values, the observed upper 95% percentiles were still within the 95% confidence intervals of the model prediction (Fig. 3). Most importantly, the estimated IgG concentration of 11.1 mg/mL (or g/L) in the absence of treatment was in the range of expected endogenous IgG levels for patients with MMN.^2^, ^16^

### Model‐based simulations of clinical scenarios

**Figure 4:**
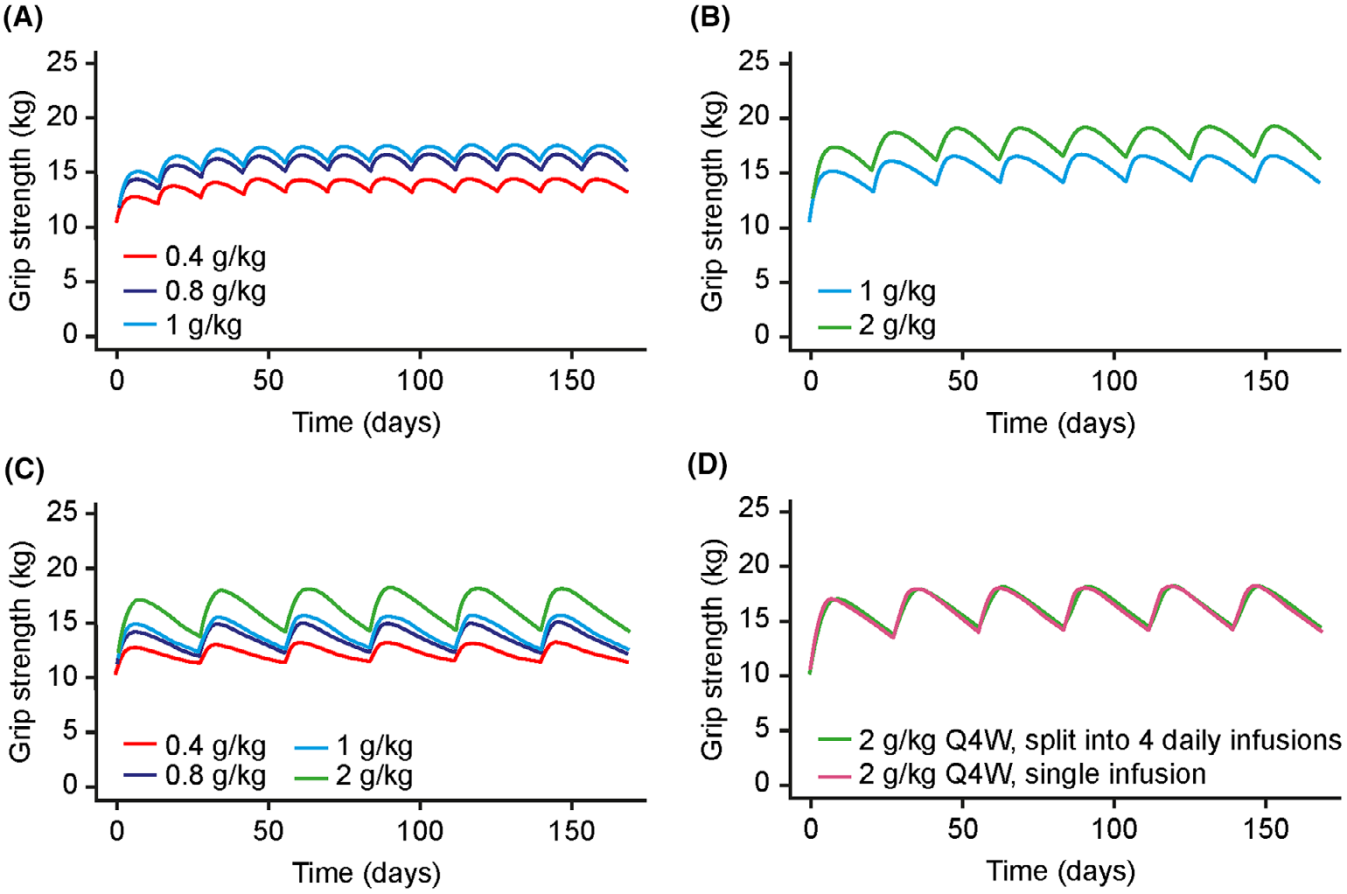
Simulated profiles of grip strength with IVIG 10% treatment (A) every 2 weeks, (B) every 3 weeks, (C) every 4 weeks, and (D) 2 g/kg split into four infusions every 4 weeks. IVIG, intravenous immunoglobulin.

**Figure 5:**
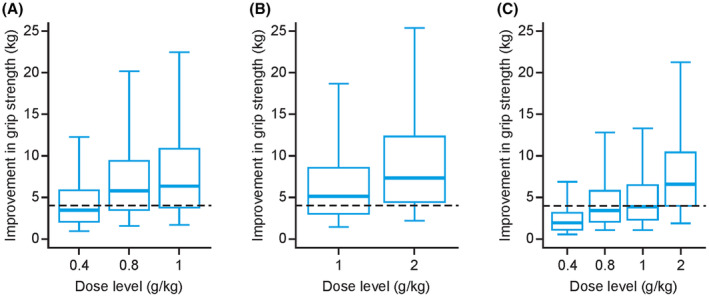
Individual improvements in average grip strength (5–95% intervals) during IVIG 10% treatment at steady‐state when administered every (A) 2 weeks, (B) 3 weeks, and (C) 4 weeks. Dashed line represents the hypothetical minimum clinically meaningful threshold of 4 kg. Central bold lines reflect median values. IVIG, intravenous immunoglobulin.

Steady‐state grip strength was shown to increase with rising dose for the Q2W, Q3W, and Q4W dosing intervals (Figs. 4 and 5). Peaks in grip strength after IgG infusion were followed by decreases over the course of the dosing interval across all simulated clinical scenarios, with trough grip strength values more strongly trending toward median GBASE values with increasing length of the treatment interval. As an example, for 1 g/kg doses, median grip strength values at trough were 16.2 kg with Q2W dosing (median GBASE value 10.5 kg), 14.2 kg with Q3W dosing (median GBASE 10.5 kg), and 12.4 kg with the Q4W dosing scenario (median GBASE 10.2 kg; Table 2). Therefore, some patients who respond to treatment may experience a deterioration in grip strength toward the end of the Q4W inter‐dose interval. Improvement in median grip strength for the more affected hand varied substantially between individuals, 5–95% range: 1.0–22.5 kg with Q2W dosing, 1.4–25.5 kg with Q3W dosing, and 0.5–21.3 kg with Q4W dosing (Table 2).

**Table 2:** Simulated grip strength outcomes using the final PK–PD model for 2‐weekly, 3‐weekly, and 4‐weekly dosing scenarios.

Treatment scenario	GBASE, median (5–95%)	GS_AV,SS_, median (5–95%)	Trough GS, median (5–95%)	GS improvement, median (5–95%)	Patients with >4 kg GS improvement, n (%)
2‐weekly					
0.4 g/kg	10.6 (3.1, 36.2)	14.1 (4.0, 46.5)	13.4 (3.8, 44.3)	3.5 (1.0, 12.2)	444 (44.4)
0.8 g/kg	10.4 (3.0, 36.6)	16.1 (4.6, 57.3)	15.3 (4.4, 54.2)	5.8 (1.6, 20.2)	697 (69.7)
1 g/kg	10.5 (2.8, 35.2)	17.0 (4.6, 56.6)	16.2 (4.3, 53.6)	6.4 (1.7, 22.5)	722 (72.2)
3‐weekly					
1 g/kg	10.5 (3.0, 36.6)	15.7 (4.4, 54.9)	14.2 (4.0, 49.8)	5.0 (1.4, 18.7)	615 (61.5)
2 g/kg	10.3 (3.2, 35.0)	17.5 (5.5, 61.4)	15.9 (4.9, 55.7)	7.2 (2.2, 25.5)	788 (78.8)
4‐weekly					
0.4 g/kg	10.2 (3.0, 36.9)	12.2 (3.6, 44.4)	11.1 (3.3, 40.8)	1.9 (0.5, 6.9)	167 (16.7)
0.8 g/kg	10.8 (3.3, 39.1)	14.1 (4.3, 51.6)	12.5 (3.8, 45.9)	3.4 (1.1, 12.8)	423 (42.3)
1 g/kg	10.2 (3.0, 33.5)	14.1 (4.1, 46.6)	12.4 (3.6, 40.9)	3.9 (1.1, 13.3)	481 (48.1)
2 g/kg	11.2 (3.2, 35.0)	17.9 (5.1, 55.8)	15.5 (4.5, 48.2)	6.6 (1.8, 21.3)	745 (74.5)

All measures are in kg, unless otherwise stated; (5–95%) represents the 5th and 95th percentiles around the median.

GBASE, latent model‐derived parameter of grip strength in the absence of treatment; GS, grip strength; GS_AV,SS_, average grip strength at steady‐state; PK–PD, pharmacokinetic–pharmacodynamic.

With Q2W dosing, the median (5–95% range) predicted improvement of grip strength of the more affected hand was 3.5 kg (1.0–12.2 kg) at a dose level of 0.4 g/kg, 5.8 kg (1.6–20.2 kg) at a dose level of 0.8 g/kg and 6.4 kg (1.7–22.5 kg) at a dose level of 1 g/kg (Fig. 5). Simulations showed that 0.4, 0.8, and 1 g/kg IVIG 10% doses Q2W resulted in 44.4%, 69.7%, and 72.2% of patients achieving a clinically significant increase in grip strength (>4 kg), respectively.

For the Q3W dosing regimen, the median (5–95% range) predicted improvement of grip strength for the more affected hand was 5.0 kg (1.4–18.7 kg) at a dose level of 1 g/kg and 7.2 kg (2.2–25.5 kg) at a dose level of 2 g/kg, with 1 and 2 g/kg IVIG 10% doses Q3W resulting in 61.5% and 78.8% of patients achieving a clinically significant increase in grip strength, respectively (Fig. 5).

Finally, with Q4W dosing, the median (5–95% range) predicted improvement of grip strength for the more affected hand was 1.9 kg (0.5–6.9 kg) at a dose level of 0.4 g/kg, 3.4 kg (1.1–12.8 kg) at a dose level of 0.8 g/kg, 3.9 kg (1.1–13.3 kg) at a dose level of 1 g/kg and 6.6 kg (1.8–21.3 kg) at a dose level of 2 g/kg (Fig. 5). According to these simulations, 0.4, 0.8, 1, and 2 g/kg IVIG 10% doses Q4W resulted in 16.7%, 42.3%, 48.1%, and 74.5% of patients achieving a clinically significant increase in grip strength, respectively.

## Discussion

MMN is a rare chronic immune‐mediated neuropathy with impaired grip strength representing a common disease symptom.^4^ While IVIG 10% has been shown to be an effective treatment for MMN,^7^, ^8^ little is known regarding the interplay between IVIG 10% dose, serum IgG levels, and clinical responses in affected patients.^6^, ^14^, ^15^, ^16^ Characterization of these relationships will help to understand how patients respond to IVIG treatment and also aid the optimization of doses and treatment regimens for patients with the disease.^18^, ^19^ The population PK–PD model developed in this study is the first to describe serum IgG PK and grip strength PD profiles, as well as their relationship following IVIG 10% administration in patients with MMN, using serum trough concentrations of total IgG and grip strength data from a prior randomized Phase 3 study.^1^

At study entry, the grip strength of both the more affected and less affected hands varied widely, with an associated exploratory analysis also showing no correlation between serum IgG concentration and grip strength. Variation was also observed in IgG trough values, with some patients showing higher IgG trough levels versus baseline following placebo. This could be owing to natural fluctuation of IgG levels due to changes in disease status and endogenous IgG production. Despite variation in grip strength and IgG trough levels, there was a correlation where an increase or decrease of a patient's serum IgG level would result in a corresponding increase or decrease in grip strength in the same direction. These results demonstrate the underlying connection between serum IgG concentration and grip strength, where treatment effect responds to fluctuation in IgG levels, rather than to an IgG trough threshold as in primary immunodeficiencies. This highlights the importance of determining the effective IVIG 10% dose to provide stable serum IgG levels on an individual patient basis.

The final population PK–PD model described the observed study data well, with acceptable goodness‐of‐fit plots and concordance of model predictions and observed data in simulated VPCs. During model development, it was noted that changes in grip strength following IVIG 10% treatment do not occur instantly with an increase in IgG concentration, but rather in a more gradual manner, which would be anticipated from a physiological perspective, although further study is needed.^2^ Based on the model, approximately 30 h would be required to achieve a 50% change in grip strength following an increase in IgG concentration (ln (2)/DTR = 30 h). This is consistent with findings from the exploratory analysis, where changes in grip strength and in serum IgG concentration were correlated when the prior study visit was used as the baseline. The maximum possible increase in grip strength suggested by the model was approximately 2.5‐fold. However, to achieve at least 95% of this improvement, the predicted total IgG concentrations would need to be in excess of 170 g/L according to the model, which would be unrealistic considering the approximate 10–40 g/L range of serum IgG that was observed in the Phase 3 study dataset and the doses of IgG generally used in MMN (recommended doses are 0.5–2.4 g/kg/month for the disease).^1^, ^7^, ^8^ This could be a result of a combination of high inter‐patient variability in grip strength and associated improvement, the lack of IgG data beyond trough levels, and the inherent limitations of the study restricting the predictive power of the model. Future availability of more comprehensive IgG and clinical response data may facilitate improved understanding of the relationship with grip strength.

Simulations using the final population PK–PD model showed that regimens with a monthly IVIG 10% dosage of at least 1.6 g/kg (0.8–1 g/kg Q2W, 2 g/kg Q3W, or 2 g/kg Q4W) could achieve a clinically significant average improvement of 4 kg in grip strength in at least 70% of patients. More frequent dosing led to smaller fluctuations in grip strength and more stable IgG concentration, which may help to reduce the magnitude of fluctuations in trough IgG levels and alleviate symptom worsening toward the end of longer dosing intervals. In addition, splitting the dose over several days appeared to have no discernable impact on grip strength and did not markedly impact the PK concentration–time profiles of IVIG 10%. Given that patients were on a stable treatment regimen, it is not unexpected that splitting a high dose of 2 g/kg Q4W over four daily infusions versus a single infusion resulted in comparable effects on grip strength. It should be noted that Q4W dosing may be more popular with younger, more active patients given the reduced frequency of infusion and associated patient burden, meaning a balance between patient preference and therapeutic efficacy is required. Our findings suggest that dosing regimens can be tailored on an individual basis to improve patient comfort and maintain IgG levels above individual protective thresholds, if established, dependent on the patient's tolerance for grip strength fluctuations, clinical needs, and the convenience regarding frequency of infusions. Similarly, a degree of flexibility to adapt dosing regimens to the needs of individual patients was demonstrated by a modeling study of subcutaneous immunoglobulin in CIDP, which showed that dosing regimens from daily to Q2W resulted in exposure equivalent to that of weekly dosing.^27^ While there is limited information in the literature regarding grip strength in patients with MMN, a meta‐analysis of four randomized, double‐blind, placebo‐controlled trials (*n* = 34) reported that 78% of patients achieved a significant improvement in muscle strength outcomes following IVIG treatment (assessed using a range of objective measures or reported subjectively by patients) compared with a 4% increase for placebo.^1^, ^33^ This reflects the modeling results reported here for dosing regimens of ≥1.6 g/kg/month.

Strengths of this analysis include that it is the first to comprehensively evaluate the connection between serum IgG level and the representative clinical efficacy outcome for MMN, the first published PK–PD model in MMN that considers the interplay between endogenous and exogenous IgG, and that both grip strength and IgG PK following IVIG 10% administration were described adequately by the final model. The model may also support the design and facilitation of future clinical investigations of a range of immunoglobulin therapies, as well as guide treatment decisions with regard to various dose ranges and regimens in real‐world clinical settings for individual patients.

There are also several limitations to this analysis that should be considered, some inherent to the design of the Phase 3 study from which the source data were extracted. To begin with, as all patients in the Phase 3 study were on stable and active IVIG treatment on study entry, no observations of grip strength or serum IgG in the absence of treatment (i.e., immunoglobulin‐naïve patients) were available although patients with MMN are unlikely to be able to cease treatment for a prolonged period to provide a more representative baseline. In addition, as aforementioned, the model was based on trough IgG observations only, as no IgG observations were collected around the time of maximum plasma concentrations, or at any other intermediate time between doses in the Phase 3 study. Moreover, the study only included 12 women in the overall population of 44 patients, meaning gender‐related differences could not be conclusively assessed. Some parameter estimates were not precise, limiting the accuracy of predictions that could be made using the model. Nonetheless, the final model described the observed data well (Fig. 3).

The use of innovative designs in future studies, including the optimization of PK sampling, will be key in improving our understanding of dose–exposure–response relationships in patients with MMN receiving IgG treatment, and the inclusion of data from immunoglobulin‐naïve patients, studies with more comprehensive PK sampling, and greater numbers of female patients with MMN in future iterations of the model may help to increase its precision. It is hoped that this population PK–PD model may also be adapted to study the relationship between serum IgG levels and clinical efficacy outcomes in similar patient populations, such as those with chronic inflammatory demyelinating polyradiculoneuropathy. In addition, it may be beneficial for a future study to assess whether strategies to reduce the magnitude of fluctuations in serum trough IgG levels, such as more frequent dosing (e.g., every 2 weeks vs. every 4 weeks), are related to improved outcomes for patients with MMN.

In conclusion, the dose–exposure–efficacy response relationships for IVIG 10% have been well‐characterized in this study, with the indirect response population PK–PD linked model successfully describing the relationship between IVIG 10% dose, IgG serum total trough levels, and grip strength in patients with MMN. Model‐based simulations indicated that a dosing regimen of ≥1.6 g/kg/month (e.g., 0.8–1 g/kg every 2 weeks and 2 g/kg every 3–4 weeks) would achieve clinically meaningful improvements in grip strength in at least 70% of patients, and that more frequent dosing may maintain a more consistent response. Splitting the dose over multiple days for high dose levels (>1 g/kg) in one dosing course is expected to have no significant or clinically meaningful impact on grip strength, meaning dosing regimens may be tailored on an individual basis for improved patient comfort, clinical outcomes, and convenience. Overall, this population PK–PD model represents a useful tool that will allow further investigation of the dose–exposure–efficacy response relationship in similar patient populations or subpopulations of interest. It is hoped that the model may also be used to support the design of future clinical trials of immunoglobulin therapies and aid clinicians in optimization of dosing regimens in real‐world clinical settings for individual patients with MMN.

## Author Contributions

All authors provided substantial contributions to the conception or design of the work; or the acquisition, analysis, or interpretation of data for the work. All authors contributed to the drafting of the work or revising it critically for important intellectual content. All authors provided final approval of the version to be published. All authors agreed to be accountable for all aspects of the work in ensuring that questions related to the accuracy or integrity of any part of the work are appropriately investigated and resolved.

## Funding Information

The study was sponsored by Takeda Development Center Americas, Inc.

## Conflict of Interest

ZL is an employee of Takeda Development Center Americas, Inc. and is a Takeda shareholder. SR is an employee of Cognigen, a division of Simulations Plus, which acted as a consultant to Takeda Development Center Americas, Inc. during this study. RF was an employee of Cognigen, a division of Simulations Plus at the time of the study. LY was an employee of Takeda Development Center Americas, Inc. at the time of the study and is a Takeda shareholder.

## Supporting information


Appendix S1.
