# Supplementary material for: Dose–exposure–efficacy response of intravenous immunoglobulin G 10% in multifocal motor neuropathy
Source: Ann Clin Transl Neurol. 2024 Jul 8;11(8):1977–87. doi: 10.1002/acn3.52098 (PMC11330225; doi:10.1002/acn3.52098)
Supplement: Supplementary file 1 — Appendix S1. [file ACN3-11-1977-s001.docx]

# Supplementary Material

# Dose–exposure–efficacy response of intravenous immunoglobulin G 10% in multifocal motor neuropathy

Zhaoyang Li, PhD, Stefan Roepcke, PhD, Ryan Franke, PhD & Leman Yel, MD

## Supplementary Methods

Analytic software: All data analyses and presentations were performed using SAS version 9.4 (SAS Institute Inc., Cary, NC, USA),^1^ R version 3.4.3 (R Foundation, Vienna, Austria)^2^ and KIWI version 4 (Cognigen, a division of Simulations Plus, Buffalo NY, USA).^3^

**References**

1. SAS Institute Inc. SAS [computer program]. Current version available at https://www.sas.com/en_gb/home.html

2. R Foundation. R [computer program]. Current version available at https://www.r-project.org/

3. Cognigen (a division of Simulations Plus). KIWI [computer program]. Current version https://www.simulations-plus.com/resource/cognigen-launches-kiwi-version-4-0/

## Supplementary Table 1. Parameter estimates for the final population PK–PD model of IVIG 10% and grip strength.

| Parameter | | Final parameter estimate | | Magnitude of variability | |
| --- | --- | --- | --- | --- | --- |
|  |  | Population mean | %RSE | Final estimate | %RSE |
| V1 | Volume of distribution (mL) | 6480 | 8.4 | 18.5%CV | 47.3 |
|  | Exponent of (LBM/56.54) for V1 | 2.17 | 15.7 |  |  |
| CBASE | IgG concentration in the absence of treatment (mg/mL) | 11.1 | 3.2 | 18.0%CV | 19.8 |
| K_EL_ | Elimination rate (1/hour) | 0.00243 | 7.3 | NE | NA |
| GBASE | Grip strength in the absence of treatment (kg) | 10.6 | 12.8 | 86.8%CV | 28.8 |
| DTR | Deterioration rate (1/hour) | 0.0230 | 88.0 | NE | NA |
| C_50_ | IgG concentration for 50% inhibition (mg/mL) | 9.41 | FIXED | NE | NA |
| LIMAX | Logit transformed maximum inhibitory effect of grip strength deterioration | 0.433 | 83.1 | NE | NA |
| RV for pharmacokinetics | | 0.00905 | 17.1 | 9.5%CV | NA |
| RV for pharmacodynamics | | 0.0602 | 24.3 | 24.5%CV | NA |

Shrinkage estimates: 33.5% for IIV in V1, 6.9% for IIV in CBASE, 0.8% for IIV in GBASE, 9.3% for RV of pharmacokinetics, 7.0% for RV of pharmacodynamics.

%CV, coefficient of variation; C_50_, concentration at which 50% of the maximum inhibitory effect is achieved; CBASE, latent model-derived parameter for IgG concentration in the absence of treatment; DTR, deterioration rate of grip strength; GBASE, latent model-derived parameter of grip strength in the absence of treatment; IgG, immunoglobulin G; IIV, interindividual variability; IVIG, intravenous immunoglobulin; K_EL_, elimination rate constant; LBM, lean body mass; LIMAX, logit transformed maximum inhibitory effect, where the maximum inhibitory effect (IMAX) = exp(LIMAX)/(1+exp(LIMAX)); NA, not applicable; NE, not evaluated; PK–PD, pharmacokinetic–pharmacodynamic; RSE, relative standard error; RV, residual variability; V1, volume of distribution.

##
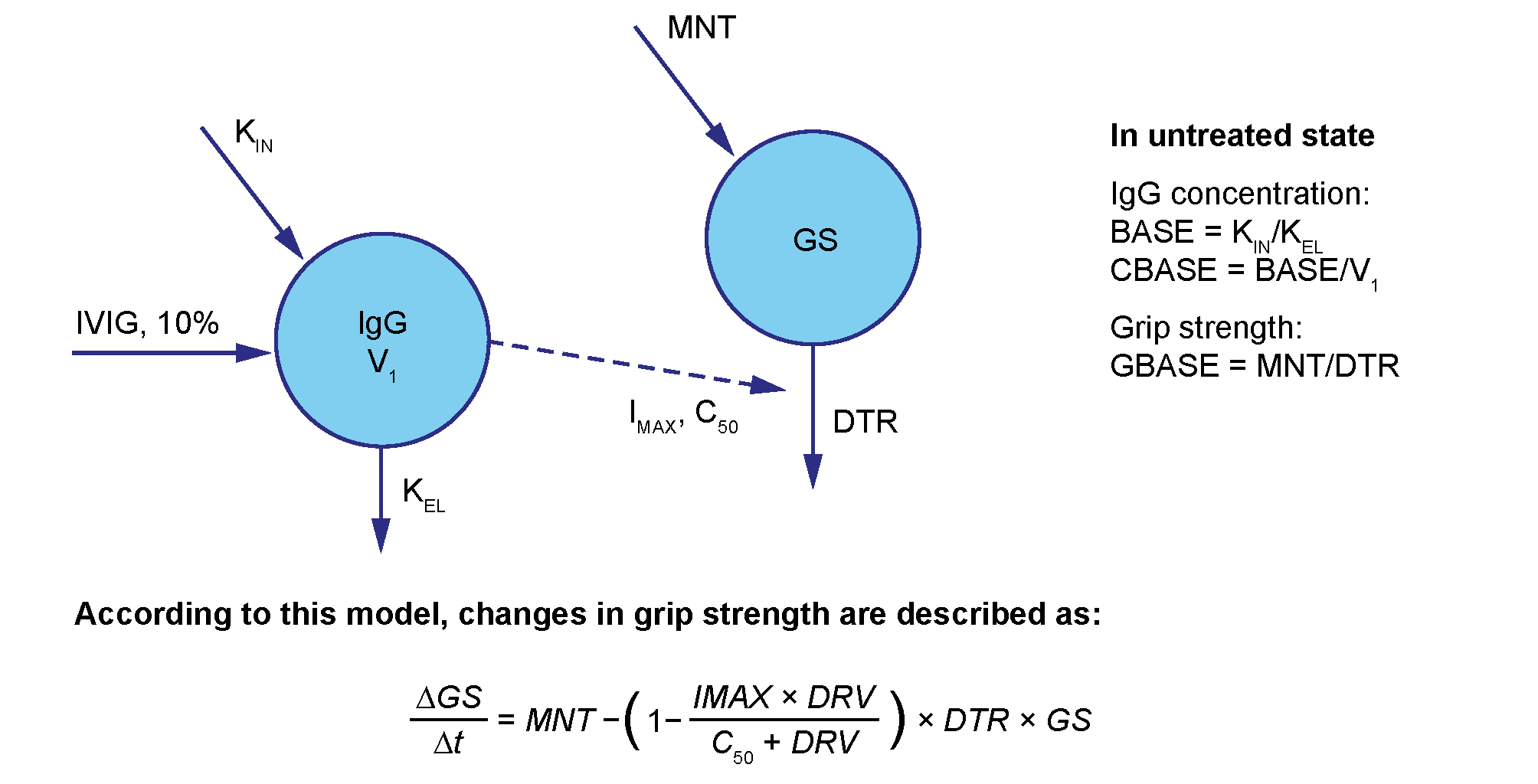
**Supplementary Figure 1.** Schematic of the final indirect response PK–PD model for grip strength. BASE, latent model-derived parameter of IgG amount in the absence of treatment; C_50_, concentration at which 50% of the maximum inhibitory effect is achieved; CBASE, latent model-derived parameter of IgG concentration in the absence of treatment; DRV, IgG concentration in excess of baseline IgG concentration; DTR, deterioration rate of grip strength; GBASE, latent model-derived parameter of grip strength in the absence of treatment; GS, grip strength; ΔGS, change in grip strength; IgG, immunoglobulin G; IMAX, maximum inhibitory effect; IVIG, intravenous immunoglobulin; K_IN_, endogenous production rate of IgG; K_EL_, elimination rate of IgG; MNT, production rate of grip strength; PK–PD, pharmacokinetic–pharmacodynamic; Δt, change in time; V1, volume of distribution.

**
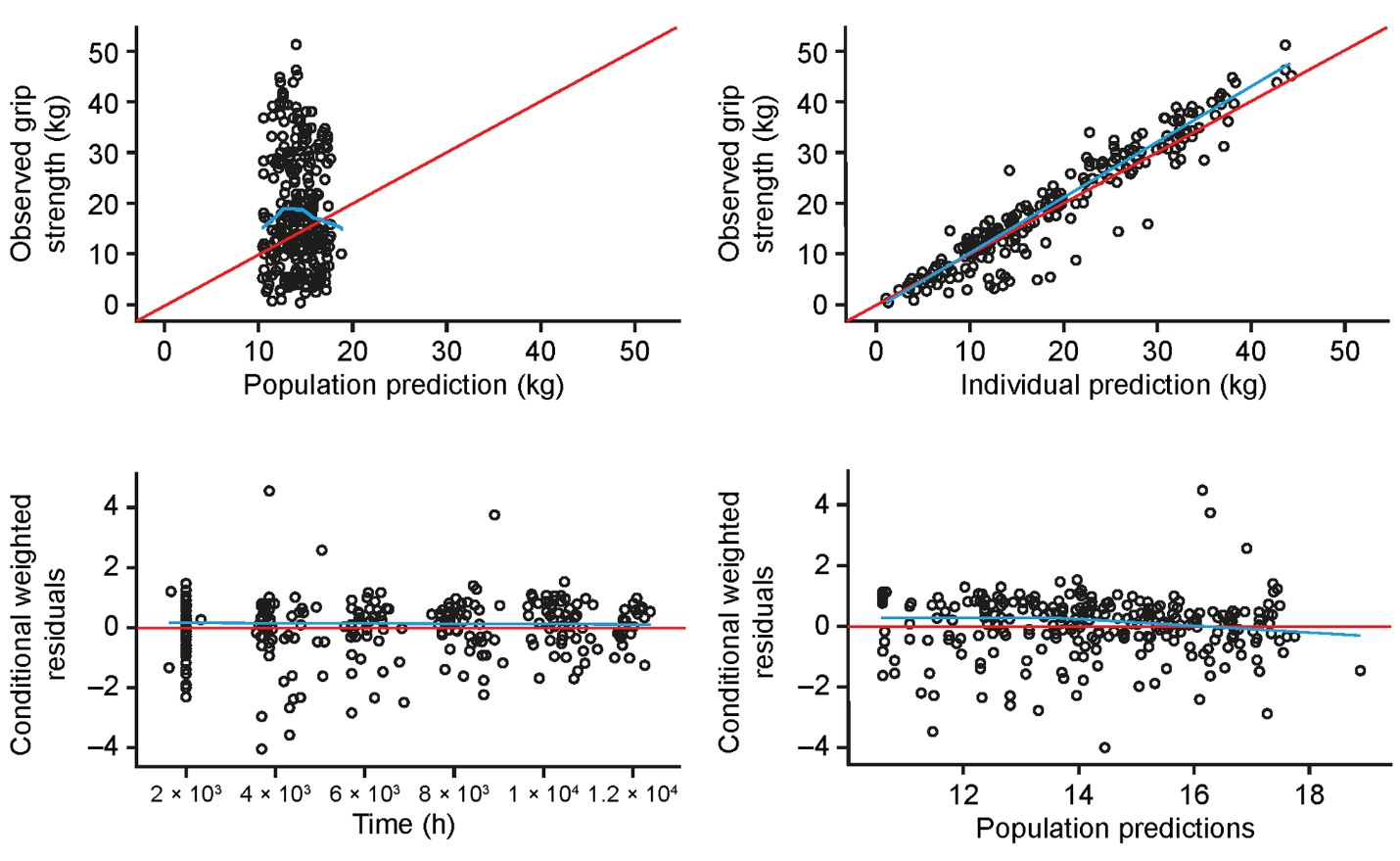
**

## Supplementary Figure 2. Goodness-of-fit plots for the final population PK–PD model. Dots represent individual data points, blue lines represent smoothed curve based on the datapoints, and red lines represent no change (Δ = 0). PK–PD, pharmacokinetic–pharmacodynamic.
